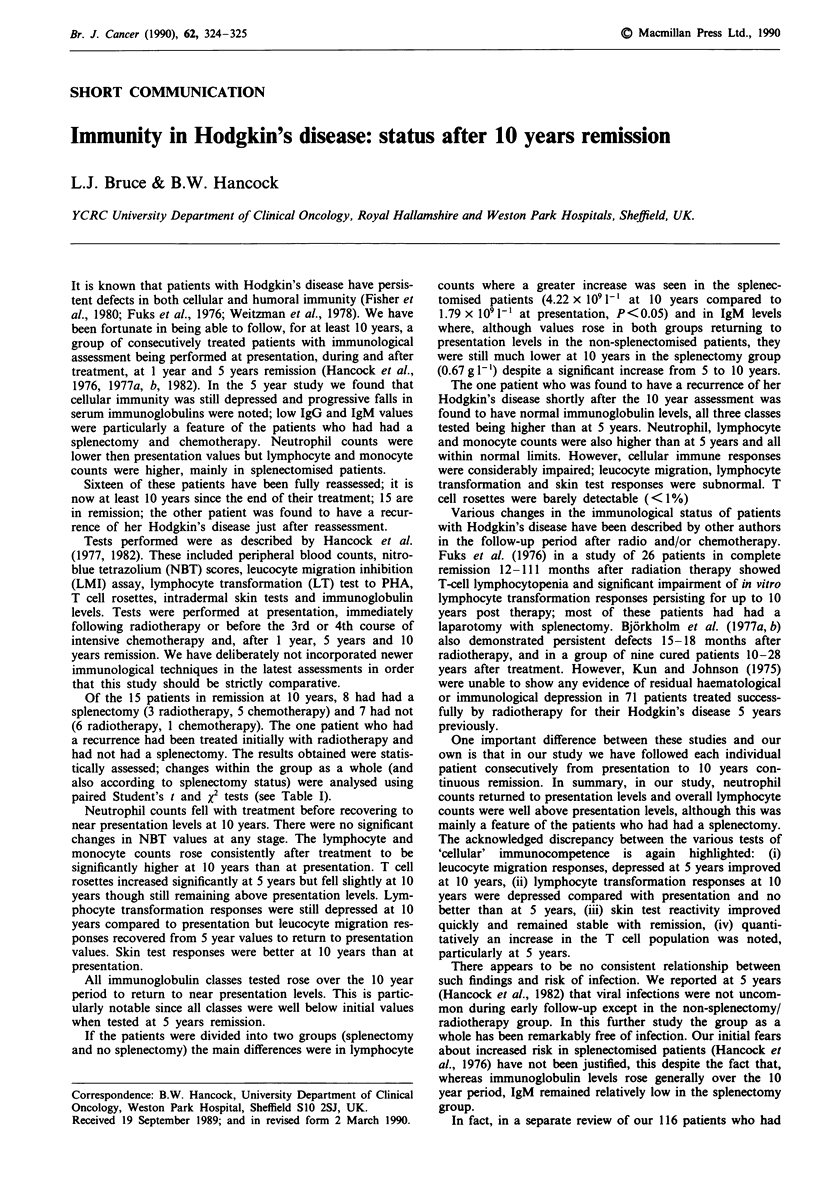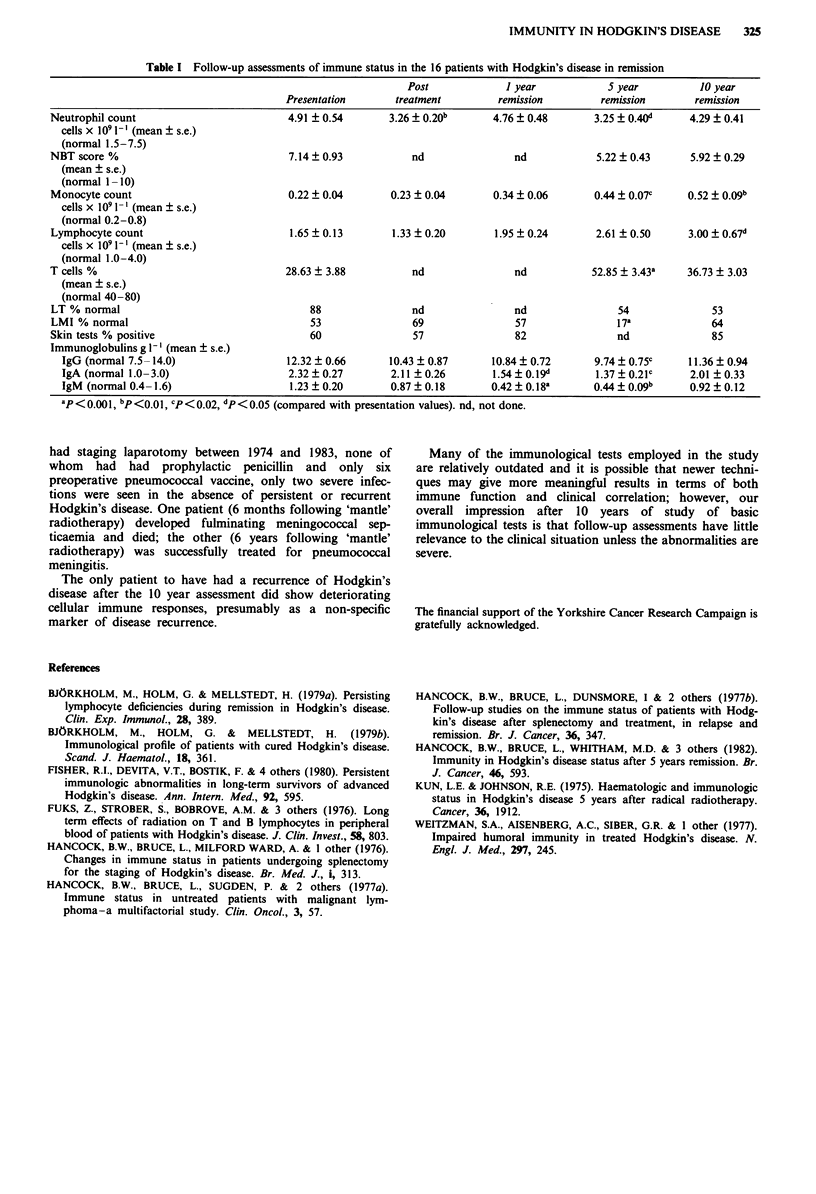# Immunity in Hodgkin's disease: status after 10 years remission.

**DOI:** 10.1038/bjc.1990.287

**Published:** 1990-08

**Authors:** L. J. Bruce, B. W. Hancock

**Affiliations:** YCRC University Department of Clinical Oncology, Royal Hallamshire Hospital, Sheffield, UK.


 © Macmillan Press Ltd., 1990

SHORT COMMUNICATION

### Immunity in Hodgkin's disease: status after 10 years remission

L.J. Bruce & B.W. Hancock

*YCRC University Department of Clinical Oncology, Royal Hallamshire and Weston Park Hospitals, Sheffield, UK.*

It is known that patients with Hodgkin's disease have persistent defects in both cellular and humoral immunity (Fisher *et al.*, 1980; Fuks *et al.*, 1976; Weitzman *et al.*, 1978). We have been fortunate in being able to follow, for at least 10 years, a group of consecutively treated patients with immunological assessment being performed at presentation, during and after treatment, at 1 year and 5 years remission (Hancock *et al.*, 1976, 1977*a*, *b*, 1982). In the 5 year study we found that cellular immunity was still depressed and progressive falls in serum immunoglobulins were noted; low IgG and IgM values were particularly a feature of the patients who had had a splenectomy and chemotherapy. Neutrophil counts were lower then presentation values but lymphocyte and monocyte counts were higher, mainly in splenectomised patients.

Sixteen of these patients have been fully reassessed; it is now at least 10 years since the end of their treatment; 15 are in remission; the other patient was found to have a recurrence of her Hodgkin's disease just after reassessment.

Tests performed were as described by Hancock *et al.* (1977, 1982). These included peripheral blood counts, nitroblue tetrazolium (NBT) scores, leucocyte migration inhibition (LMI) assay, lymphocyte transformation (LT) test to PHA, T cell rosettes, intradermal skin tests and immunoglobulin levels. Tests were performed at presentation, immediately following radiotherapy or before the 3rd or 4th course of intensive chemotherapy and, after 1 year, 5 years and 10 years remission. We have deliberately not incorporated newer immunological techniques in the latest assessments in order that this study should be strictly comparative.

Of the 15 patients in remission at 10 years, 8 had had a splenectomy (3 radiotherapy, 5 chemotherapy) and 7 had not (6 radiotherapy, 1 chemotherapy). The one patient who had a recurrence had been treated initially with radiotherapy and had not had a splenectomy. The results obtained were statistically assessed; changes within the group as a whole (and also according to splenectomy status) were analysed using paired Student's $t$ and $\chi^2$ tests (see Table I).

Neutrophil counts fell with treatment before recovering to near presentation levels at 10 years. There were no significant changes in NBT values at any stage. The lymphocyte and monocyte counts rose consistently after treatment to be significantly higher at 10 years than at presentation. T cell rosettes increased significantly at 5 years but fell slightly at 10 years though still remaining above presentation levels. Lymphocyte transformation responses were still depressed at 10 years compared to presentation but leucocyte migration responses recovered from 5 year values to return to presentation values. Skin test responses were better at 10 years than at presentation.

All immunoglobulin classes tested rose over the 10 year period to return to near presentation levels. This is particularly notable since all classes were well below initial values when tested at 5 years remission.

If the patients were divided into two groups (splenectomy and no splenectomy) the main differences were in lymphocyte counts where a greater increase was seen in the splenectomised patients ($4.22 \times 10^9 l^{-1}$ at 10 years compared to $1.79 \times 10^9 l^{-1}$ at presentation, $P < 0.05$) and in IgM levels where, although values rose in both groups returning to presentation levels in the non-splenectomised patients, they were still much lower at 10 years in the splenectomy group ($0.67 g l^{-1}$) despite a significant increase from 5 to 10 years.

The one patient who was found to have a recurrence of her Hodgkin's disease shortly after the 10 year assessment was found to have normal immunoglobulin levels, all three classes tested being higher than at 5 years. Neutrophil, lymphocyte and monocyte counts were also higher than at 5 years and all within normal limits. However, cellular immune responses were considerably impaired; leucocyte migration, lymphocyte transformation and skin test responses were subnormal. T cell rosettes were barely detectable ($<1\%$)

Various changes in the immunological status of patients with Hodgkin's disease have been described by other authors in the follow-up period after radio and/or chemotherapy. Fuks *et al.* (1976) in a study of 26 patients in complete remission 12–111 months after radiation therapy showed T-cell lymphocytopenia and significant impairment of *in vitro* lymphocyte transformation responses persisting for up to 10 years post therapy; most of these patients had had a laparotomy with splenectomy. Björkholm *et al.* (1977*a*, *b*) also demonstrated persistent defects 15–18 months after radiotherapy, and in a group of nine cured patients 10–28 years after treatment. However, Kun and Johnson (1975) were unable to show any evidence of residual haematological or immunological depression in 71 patients treated successfully by radiotherapy for their Hodgkin's disease 5 years previously.

One important difference between these studies and our own is that in our study we have followed each individual patient consecutively from presentation to 10 years continuous remission. In summary, in our study, neutrophil counts returned to presentation levels and overall lymphocyte counts were well above presentation levels, although this was mainly a feature of the patients who had had a splenectomy. The acknowledged discrepancy between the various tests of 'cellular' immunocompetence is again highlighted: (i) leucocyte migration responses, depressed at 5 years improved at 10 years, (ii) lymphocyte transformation responses at 10 years were depressed compared with presentation and no better than at 5 years, (iii) skin test reactivity improved quickly and remained stable with remission, (iv) quantitatively an increase in the T cell population was noted, particularly at 5 years.

There appears to be no consistent relationship between such findings and risk of infection. We reported at 5 years (Hancock *et al.*, 1982) that viral infections were not uncommon during early follow-up except in the non-splenectomy/radiotherapy group. In this further study the group as a whole has been remarkably free of infection. Our initial fears about increased risk in splenectomised patients (Hancock *et al.*, 1976) have not been justified, this despite the fact that, whereas immunoglobulin levels rose generally over the 10 year period, IgM remained relatively low in the splenectomy group.

In fact, in a separate review of our 116 patients who had

Correspondence: B.W. Hancock, University Department of Clinical Oncology, Weston Park Hospital, Sheffield S10 2SJ, UK.
Received 19 September 1989; and in revised form 2 March 1990.

**Table I** Follow-up assessments of immune status in the 16 patients with Hodgkin's disease in remission

| | *Presentation* | *Post treatment* | *1 year remission* | *5 year remission* | *10 year remission* |
|---|---|---|---|---|---|
| Neutrophil count cells × $10^9 l^{-1}$ (mean ± s.e.) (normal 1.5–7.5) | 4.91 ± 0.54 | 3.26 ± 0.20$^b$ | 4.76 ± 0.48 | 3.25 ± 0.40$^d$ | 4.29 ± 0.41 |
| NBT score % (mean ± s.e.) (normal 1–10) | 7.14 ± 0.93 | nd | nd | 5.22 ± 0.43 | 5.92 ± 0.29 |
| Monocyte count cells × $10^9 l^{-1}$ (mean ± s.e.) (normal 0.2–0.8) | 0.22 ± 0.04 | 0.23 ± 0.04 | 0.34 ± 0.06 | 0.44 ± 0.07$^c$ | 0.52 ± 0.09$^b$ |
| Lymphocyte count cells × $10^9 l^{-1}$ (mean ± s.e.) (normal 1.0–4.0) | 1.65 ± 0.13 | 1.33 ± 0.20 | 1.95 ± 0.24 | 2.61 ± 0.50 | 3.00 ± 0.67$^d$ |
| T cells % (mean ± s.e.) (normal 40–80) | 28.63 ± 3.88 | nd | nd | 52.85 ± 3.43$^a$ | 36.73 ± 3.03 |
| LT % normal | 88 | nd | nd | 54 | 53 |
| LMI % normal | 53 | 69 | 57 | 17$^a$ | 64 |
| Skin tests % positive | 60 | 57 | 82 | nd | 85 |
| Immunoglobulins $g l^{-1}$ (mean ± s.e.) | | | | | |
| IgG (normal 7.5–14.0) | 12.32 ± 0.66 | 10.43 ± 0.87 | 10.84 ± 0.72 | 9.74 ± 0.75$^c$ | 11.36 ± 0.94 |
| IgA (normal 1.0–3.0) | 2.32 ± 0.27 | 2.11 ± 0.26 | 1.54 ± 0.19$^d$ | 1.37 ± 0.21$^c$ | 2.01 ± 0.33 |
| IgM (normal 0.4–1.6) | 1.23 ± 0.20 | 0.87 ± 0.18 | 0.42 ± 0.18$^a$ | 0.44 ± 0.09$^b$ | 0.92 ± 0.12 |

$^aP<0.001$, $^bP<0.01$, $^cP<0.02$, $^dP<0.05$ (compared with presentation values). nd, not done.

had staging laparotomy between 1974 and 1983, none of whom had had prophylactic penicillin and only six preoperative pneumococcal vaccine, only two severe infections were seen in the absence of persistent or recurrent Hodgkin's disease. One patient (6 months following 'mantle' radiotherapy) developed fulminating meningococcal septicaemia and died; the other (6 years following 'mantle' radiotherapy) was successfully treated for pneumococcal meningitis.

The only patient to have had a recurrence of Hodgkin's disease after the 10 year assessment did show deteriorating cellular immune responses, presumably as a non-specific marker of disease recurrence.

Many of the immunological tests employed in the study are relatively outdated and it is possible that newer techniques may give more meaningful results in terms of both immune function and clinical correlation; however, our overall impression after 10 years of study of basic immunological tests is that follow-up assessments have little relevance to the clinical situation unless the abnormalities are severe.

The financial support of the Yorkshire Cancer Research Campaign is gratefully acknowledged.

**References**

BJÖRKHOLM, M., HOLM, G. & MELLSTEDT, H. (1979*a*). Persisting lymphocyte deficiencies during remission in Hodgkin's disease. *Clin. Exp. Immunol.*, **28**, 389.

BJÖRKHOLM, M., HOLM, G. & MELLSTEDT, H. (1979*b*). Immunological profile of patients with cured Hodgkin's disease. *Scand. J. Haematol.*, **18**, 361.

FISHER, R.I., DEVITA, V.T., BOSTIK, F. & 4 others (1980). Persistent immunologic abnormalities in long-term survivors of advanced Hodgkin's disease. *Ann. Intern. Med.*, **92**, 595.

FUKS, Z., STROBER, S., BOBROVE, A.M. & 3 others (1976). Long term effects of radiation on T and B lymphocytes in peripheral blood of patients with Hodgkin's disease. *J. Clin. Invest.*, **58**, 803.

HANCOCK, B.W., BRUCE, L., MILFORD WARD, A. & 1 other (1976). Changes in immune status in patients undergoing splenectomy for the staging of Hodgkin's disease. *Br. Med. J.*, **i**, 313.

HANCOCK, B.W., BRUCE, L., SUGDEN, P. & 2 others (1977*a*). Immune status in untreated patients with malignant lymphoma-a multifactorial study. *Clin. Oncol.*, **3**, 57.

HANCOCK, B.W., BRUCE, L., DUNSMORE, I & 2 others (1977*b*). Follow-up studies on the immune status of patients with Hodgkin's disease after splenectomy and treatment, in relapse and remission. *Br. J. Cancer*, **36**, 347.

HANCOCK, B.W., BRUCE, L., WHITHAM, M.D. & 3 others (1982). Immunity in Hodgkin's disease status after 5 years remission. *Br. J. Cancer*, **46**, 593.

KUN, L.E. & JOHNSON, R.E. (1975). Haematologic and immunologic status in Hodgkin's disease 5 years after radical radiotherapy. *Cancer*, **36**, 1912.

WEITZMAN, S.A., AISENBERG, A.C., SIBER, G.R. & 1 other (1977). Impaired humoral immunity in treated Hodgkin's disease. *N. Engl. J. Med.*, **297**, 245.